# Impact of anti-Müllerian hormone on pregnancy outcomes in in vitro maturation: a retrospective cohort study

**DOI:** 10.1186/s13048-026-02033-w

**Published:** 2026-02-25

**Authors:** Mengjie Fan, Binbin Tu, Jing Shi, Linjing Qi, Donglin Han, Qiong Liu, Jun Zhang, Jie Zhao, Rong Li, Jie Qiao

**Affiliations:** 1 State Key Laboratory of Female Fertility Promotion, Center for Reproductive Medicine, Department of Obstetrics and Gynecology, Peking University Third Hospital, Beijing, 100191 China; 2 National Clinical Research for Obstetrics and Gynecology, Beijing, China; 3 Key Laboratory of Assisted Reproduction, Ministry of Education, Beijing, China; 4 Beijing Key Laboratory of Reproductive Endocrinology and Assisted Reproductive Technology, Beijing, China; 5 Department of Pharmacy, Peking University Third Hospital, Beijing, China

**Keywords:** Anti-Müllerian hormone, *In vitro* maturation, Pregnancy outcomes

## Abstract

**Background:**

In vitro maturation (IVM) presents a potential alternative to conventional IVF for special subgroups of infertile couples. Anti-Müllerian Hormone (AMH) is widely regarded as the most robust biomarker for assessing ovarian reserve. However, the relationship between AMH and subsequent pregnancy success rates following IVM remains poorly characterized.

**Design:**

This was a retrospective cohort study. Infertile patients undergoing IVM treatment at Peking University Third Hospital Reproductive Medicine Center between January 2016 and June 2024. Participants were stratified by AMH (ng/ml) quartiles: Group A (1 < AMH ≤ 7.77, *n* = 99), B (7.77 < AMH ≤ 11.91, *n* = 98), C (11.91 < AMH ≤ 17.53, *n* = 100), and D (AMH > 17.53, *n* = 96). The primary outcome was cumulative live birth rate.

**Results:**

While baseline characteristics (age, BMI, infertility duration/type, FSH) were comparable across groups, Group D demonstrated significantly higher immature oocyte yield (*P* < 0.001), and embryological parameters (transferable/high-quality embryos: *P* < 0.001) versus other groups. Paradoxically, Group A achieved superior clinical outcomes, including: cumulative live birth rate (48.8% vs. 29.1% vs. 37.7% vs. 21.6%, *P* = 0.009), cumulative clinical pregnancy rate (67.4% vs. 40.0% vs. 42.6% vs. 36.1%, *P* = 0.006), and clinical pregnancy rate (68.4% vs. 39.1% vs. 44.4% vs. 37.5%, *P* = 0.015). Multivariate analysis also indicated that excessively high AMH levels (> 17.53ng/ml) may be related to reduced clinical pregnancy rate compared to low AMH levels (*OR* = 0.281, 95% *CI*: 0.108–0.728, *P* = 0.009). No significant differences emerged between fresh versus frozen-thawed transfers.

**Conclusion:**

Elevated AMH levels (> 17.53ng/ml) predict enhanced follicular recruitment but paradoxically associate with diminished reproductive success compared to moderate AMH levels (1 < AMH ≤ 7.77 ng/ml) in IVM cycles.

**Supplementary Information:**

The online version contains supplementary material available at 10.1186/s13048-026-02033-w.

## Key message

Elevated AMH levels (> 17.53 ng/mL) predict higher oocyte yield but paradoxically result in lower pregnancy rates compared to moderate AMH levels (1-7.77 ng/mL) in IVM cycles.

**Supplementary Information:**

The online version contains supplementary material available at 10.1186/s13048-026-02033-w.

## Introduction

 In vitro maturation (IVM) of immature oocytes refers to the process of retrieving immature oocytes from the ovaries and culturing them in vitro to the metaphase II (MII) stage, enabling them to be fertilized before the embryos are transferred to the mother’s uterus for growth [1]. The first successful pregnancy case using IVM dates back to 1991 [2], and more than 5000 children have been born worldwide after IVM over the past 30 years [3]. Compared to conventional IVF, IVM offers a shorter treatment cycle, flexibility in timing, fewer monitoring sessions, and significantly lower medication costs, which can substantially reduce the economic burden on patients and decrease the incidence of adverse drug reactions [4]. Initially conceived as a method for preserving fertility in patients undergoing cancer treatments, IVM’s scope has expanded, especially in mitigating the chances of ovarian hyperstimulation syndrome (OHSS) after superovulation treatments in high-risk patients such as those with polycystic ovaries (PCO) and polycystic ovary syndrome (PCOS) [5]. Thanks to technological advancements in assisted reproductive laboratories in recent years, IVM has the potential to treat a range of patients with poor ovarian, oocyte donors, patients with thrombophilia, or with follicle-stimulating hormone (FSH) resistance to avoid the side-effects of an elevated estradiol level, and especially in PCOS patients [6, 7].

The primary factors influencing IVF outcomes include age and ovarian reserve [8]. Anti-Müllerian Hormone (AMH), primarily secreted by granulosa cells during early follicular development, with AFC (antral follicle count) are widely regarded as the most robust biomarker for assessing ovarian reserve function. Study consistently demonstrates a positive correlation between these two parameters: a higher number of small antral follicles in the ovaries is associated with increased AMH levels secreted by granulosa cells [9]. Beyond the diagnostic utility in ovarian reserve evaluation, AMH demonstrates significant potential as a diagnostic tool for ovarian pathologies, including adenomyosis, endometriosis, breast cancer, and ovarian cancer. Emerging evidence also suggests its therapeutic potential in mitigating oocyte loss induced by chemotherapy or radiotherapy [10–12]. Unlike other markers, AMH levels maintain consistency throughout the menstrual cycle, making them a staple in contemporary clinical evaluations [13]. Additionally, AMH offers a glimpse into the potential yield of oocytes in spontaneous cycles and plays a crucial role in the growth and maturation of follicles and oocytes [14].

Current evidence remains divided regarding the predictive value of age and serum AMH levels in in vitro fertilization (IVF)/ intracytoplasmic sperm injection procedures (ICSI) outcomes. While some studies demonstrate AMH’s superior predictive capacity for ovarian response compared to conventional biomarkers (FSH, estradiol, and inhibin B) [15–17], others contend that AMH primarily reflects quantitative oocyte retrieved rather than qualitative parameters such as embryo quality or pregnancy success [18]. This ongoing controversy underscores the need for further investigation into the complex relationship between AMH and assisted reproductive technology (ART) to optimize clinical protocols.

IVM presents a potential alternative to conventional IVF for special subgroups of infertile couples [19]. Existing evidence suggests a significant positive correlation between AMH levels and IVM outcomes [20]. However, the relationship between AMH concentrations and subsequent pregnancy success rates following IVM remains poorly characterized. To address this knowledge gap, we conducted a retrospective cohort study of 393 infertile patients undergoing IVM treatment between January 2016 and June 2024, since our center initiated AMH testing in 2016 as part of routine ovarian reserve assessment. This investigation had three primary objectives: (1) to elucidate the association between AMH levels and IVM outcomes, (2) to determine whether serum AMH concentrations correlate with both embryo yield and clinical outcomes in IVM cycles, and (3) to evaluate the predictive value of AMH for IVM success.

## Materials and methods

### Study subjects and grouping criteria

This study retrospectively analyzed characteristics and pregnancy outcomes of infertile patients undergoing IVM treatment at the Reproductive Medicine Center of Peking University Third Hospital from January 2016 to June 2024. Inclusion Criteria: (1) First IVM cycle; (2) Documented serum AMH levels measured within one year prior to oocyte retrieval; (3) Age 20 to 35 years. Exclusion Criteria: (1) Severe diminished ovarian reserve (AMH<1ng/ml); (2) Uterine abnormalities (including: congenital Müllerian anomalies, untreated intrauterine adhesions, adenomyosis, intramural fibroids (> 4 cm diameter) or submucosal fibroids); (3) Endometriosis; 4.Fertility preservation cycles for oncological indications; 5. Uncontrolled severe systemic diseases, such as systemic lupus erythematosus (SLE), antiphospholipid syndrome (APS), diabetes mellitus and metabolic syndrome; 6. Untreated hydrosalpinx; 7. Chromosomal abnormalities in either partner; 8. Incomplete clinical or laboratory data.

Participants were stratified into quartiles based on AMH distribution: Group A: AMH ≤ 25th percentile, Group B: 25th percentile < AMH ≤ median, Group C: median < AMH ≤ 75th percentile, and Group D: AMH > 75th percentile.

### IVM protocol

The IVM protocol was consistent through the study and did not involve gonadotropin administration. On menstrual cycle day 2, baseline hormone levels were measured in all patients. Transvaginal ultrasound-guided retrieval was initiated once the endometrial thickness reached at least 6 mm and the largest follicle was less than 10 mm. Immature oocytes were aspirated individually to maximize yield, and follicular fluid was collected in centrifuge tubes for laboratory processing immediately. Cumulus-oocyte complexes (COCs) were isolated and cultured in IVM medium (Sage IVM media kit, Origio, Denmark) supplemented with 0.075 U/mL human menopausal gonadotropin (Menopur, Ferring, Germany) under standard conditions (37 °C, 5% CO₂) for 24–48 h to assess maturity. All oocytes reaching metaphase II (MII) were inseminated using ICSI [21].

Zygotes were further cultured in G-1 plus medium (Vitrolife, Sweden) and transferred to G-2 plus medium (Vitrolife, Sweden) on the third day. Fertilization confirmation (normal 2 pronuclei [2PN]) was evaluated by embryologists 16–18 h post-insemination. Embryo morphology and developmental competence were graded on days 3, 5, and 6.

### Embryo quality assessment

Embryo evaluation is based on three key parameters: blastomere number, size symmetry, and cytoplasmic fragmentation. Transfer eligibility requires embryos to exhibit ≥ 4 cells with < 30% fragmentation. High-quality cleavage embryos are defined as those demonstrating ≥ 6 cells by day 3, symmetrical blastomeres, and < 20% fragmentation [22]. Blastocyst grading follows the Gardner classification system [23], which categorizes blastocyst development into six sequential stages based on blastocoel expansion and hatching status. Clinically suitable blastocysts (reaching Stages III-IV) with Gardner scores ≥3BB are prioritized for transfer.

### Embryo transfer

The highest-quality cleavage-stage embryos or blastocysts were prioritized for fresh transfer, with remaining viable embryos cryopreserved for future cycles.

In fresh embryo transfer cycles, the hormonal support regimen consisted of oral Progynova (2 mg twice daily, Delpharm Lille, Lys-Lez-Lannoy, France) initiated on the day of oocyte retrieval for estrogen supplementation, combined with luteal phase support starting on the first post-retrieval day through oral dydrogesterone (10 mg twice daily, Duphaston, Abbott, OLST, Netherlands) and vaginal progesterone slow-release gel (90 mg once daily, Crinone, Merck Serono, Watford, UK). Embryo transfer was performed on day 4 post-retrieval. For frozen-thawed embryo transfer cycles, endometrial preparation was achieved using an artificial cycle protocol. Oral Progynova at a dose of 3 mg twice daily was administered on days 1–3 of the menstrual cycle if the endometrial thickness was less than 6 mm and no ovarian cyst was observed on ultrasound. An ultrasound scan was repeated 10 days later. When the endometrial thickness reached 8 mm, 90 mg vaginal progesterone gel daily and oral dydrogesterone 20 mg twice daily were administered. Embryo/embryos was/were transferred on day 7 after progesterone administration.

Serum human chorionic gonadotrophin (hCG) was assessed at 14 days post-transfer, followed by ultrasound confirmation at 30 days for positive cases. Upon confirmation of intrauterine pregnancy (clinical pregnancy), the initial Progynova dosage was maintained for 3–4 weeks before initiating a 2-week tapering period, while luteal support was continued until 10–12 weeks of gestation.

### Variables and outcome measures

Patient demographic and clinical data were collected, including age, body mass index (BMI), type and duration of infertility, infertility etiology, antral follicle count (AFC), and FSH levels on menstrual cycle day 2, luteinizing hormone (LH), estradiol (E2), progesterone (P) measured two days prior to oocyte retrieval. Additionally, IVM outcomes were recorded: the total number of oocytes retrieved, mature oocytes (MII stage), fertilized oocytes, 2PN zygotes, and transferable embryos, good quality embryos. The maturation rate was calculated as the number of MII oocytes divided by the total number of oocytes retrieved. The fertilization rate was defined as the proportion of MII oocytes that displayed two pronuclei (2PN). The cleavage rate was calculated as the number of embryos that had cleaved to at least the 2-cell stage by day 2, divided by the number of normally fertilized (2PN) zygotes. The transferable embryo rate was calculated as the number of transferable embryos divided by the number of normally fertilized embryos, while the high-quality embryo rate was determined by the number of high-quality embryos divided by the number of cleaved embryos derived from 2PN zygotes.

The primary outcome of this study was the cumulative live birth rate (CLBR), defined as the occurrence of live birth following the transfer of all the embryos derived from a single oocyte retrieval cycle, excluding those who still have frozen embryos. Secondary outcomes included: clinical pregnancy rate, live birth rate, miscarriage rate in the first cycle and cumulative clinical pregnancy rate (CCPR) from the index retrieval cycle. For clarity, clinical pregnancy was confirmed either by ultrasound visualization of an intrauterine gestational sac or pathological identification of pregnancy tissue; live birth was defined as delivery of a viable infant at ≥ 28 weeks or with birth weight > 1000 g; and miscarriage referred to pregnancy loss before 28 weeks’ gestation.

### Statistical analysis

Statistical analyses were performed using SPSS software (version 22.0, IBM Corp.). Continuous variables following a normal distribution were expressed as mean ± standard deviation (SD), and comparisons were conducted using one-way ANOVA. For non-normally distributed variables, data were reported as median (25th percentile, 75th percentile), and non-parametric tests (Mann-Whitney U) were applied. Categorical variables were summarized as frequencies (percentages), and group differences were evaluated using the Chi-square (χ²) test or Fisher’s exact test, as appropriate. A two-tailed *P*-value < 0.05 was considered statistically significant. For variables showing significant differences in univariate analysis, post-hoc comparisons were performed to identify specific group disparities, and a *P* value < 0.008 was defined as indicating statistical significance. Additionally, multivariate logistic regression was employed to adjust for potential confounding factors.

## Results

### Demographic and clinical features

From January 2016 to June 2024, a total of 544 IVM cycles were initially screened. Ultimately, 393 cycles met the inclusion criteria and were enrolled in this study. Among these, 301 cases (76.6%) were diagnosed with PCOS, while the remaining 92 cases (23.4%) had infertility due to male factors, tubal factors, or unexplained causes.

The enrolled patients had a mean anti-Müllerian hormone (AMH) level of 13.30 ng/mL (range: 1.47–43.81 ng/mL). The 25th percentile, median, and 75th percentile AMH values were 7.77 ng/mL, 11.91 ng/mL, and 17.53 ng/mL, respectively. Based on these quartiles, patients were stratified into four groups: Group A: 1 < AMH ≤ 7.77 (*n* = 99), Group B: 7.77 < AMH ≤ 11.91 (*n* = 98), Group C: 11.91 < AMH ≤ 17.53 (*n* = 100), and Group D: AMH > 17.53 (*n* = 96).

The four groups showed comparable baseline characteristics including age, BMI, duration of infertility, infertility type, and pre-retrieval LH/E2 levels (all *P* > 0.05), with the exception of significantly lower basal FSH in Group D versus other groups (*P* < 0.05). We observed two distinct AMH-dependent trends: first, the proportion of infertility cases attributed to ovulatory disorders progressively increased with higher AMH levels; second, AFC demonstrated a stepwise elevation across groups (Group A: 21.70 ± 10.46; Group B: 26.10 ± 11.04; Group C: 30.77 ± 12.26; Group D: 34.67 ± 18.40), with Group D exhibiting the highest AFC. Complete data are presented in Table 1, with detailed pairwise comparisons provided in Supplementary Table 1. The median cycle day for Oocyte Pick-Up (OPU) in the study was day 11.

**Table 1:** Comparison of basic information of patients

	Group A (*n* = 99)	Group B (*n* = 98)	Group C (*n* = 100)	Group D (*n* = 96)	F/x^2^	*P* value
	1 < AMH ≤ 7.77	7.77 < AMH ≤ 11.91	11.91 < AMH ≤ 17.53	AMH > 17.53		
Age (years)	30.61 ± 3.67	30.45 ± 4.06	30.29 ± 2.82	29.37 ± 3.27	2.476	0.061
BMI (kg/m^2^)	25.07 ± 4.73	25.87 ± 5.33	25.55 ± 4.00	24.40 ± 4.00	1.904	0.128
Antral follicle count	21.70 ± 10.46	26.10 ± 11.04	30.77 ± 12.26	34.67 ± 18.40	15.414	< 0.001
Duration of infertility (years)	3.00(2.00,6.00)	3.00(2.00,5.00)	3.00(1.25,5.00)	3.00(1.00,5.00)	2.374	0.498
Type of infertility					2.669	0.466
Primary	77.8(77/99)	76.5(75/98)	70.0(70/100)	79.2(76/96)		
Secondary	22.2(22/99)	23.5(23/98)	30.0(30/100)	20.8(20/96)		
Causes of infertility						
Ovulatory dysfunction					22.348	< 0.001
Yes	61.6(61/99)	73.5(72/98)	84.0(84/100)	87.5(84/96)		
No	38.4(38/99)	26.5(26/98)	16.0(16/100)	12.5(12/96)		
Tubal factor					8.076	0.044
Yes	30.3(30/99)	30.6(30/98)	20.0(20/100)	16.7(16/96)		
No	69.7(69/99)	69.4(68/98)	80.0(80/100)	83.3(80/96)		
Male factor					1.130	0.770
Yes	42.4(42/99)	35.7(35/98)	38.0(38/100)	36.5(35/96)		
No	57.6(57/99)	64.3(63/98)	62.0(62/100)	63.5(61/96)		
Basal FSH levels (mIU/ ml)	5.62(4.54,6.65)	5.80(4.56,6.68)	5.48(4.43,6.66)	4.96(4.08,5.89)	9.274	0.026
Serum LH levels prior to oocyte retrieval (mIU/ ml)	6.79(4.08,10.67)	6.04(3.77,9.38)	7.27(3.97,9.89)	7.91(4.14,9.78)	2.491	0.477
Serum E_2_ levels prior to oocyte retrieval (pmol/ L)	175.00(131.00,239.00)	193.00(147.25,264.50)	202.00(155.50,340.50)	185.50(137.75,392.00)	6.025	0.110

### Oocyte and embryo outcomes

The analysis revealed significant AMH-dependent variations in laboratory outcomes. Group A (lowest AMH) demonstrated consistently inferior performance across all parameters, showing the lowest of immature oocytes retrieved, transferable embryos, and high-quality embryos (all *P* < 0.008 vs. Group D). Conversely, Group D (highest AMH) achieved optimal results for all measured outcomes. However, the maturation, fertilization and cleavage rate were comparable similar across the two groups.

Regarding transferable embryo rate (44.1%, 43.5%) and high-quality embryo rate (38.3%, 38.7%), they were similar between Groups C and D (*P* > 0.05), which were both higher than those in Groups A and B. Detailed numerical results are presented in Table 2, with comprehensive post-hoc comparisons provided in Supplementary Table 1.

**Table 2:** Comparative analysis of retrieved oocytes and embryo characteristic

	Group A	Group B	Group C	Group D	F/x2	*P* value
No. of oocytes retrieved	9.00(6.00,15.00)	13.00(9.00,19.00)	15.50(10.25,23.00)	20.00(14.00,28.75)	67.791	< 0.001
Maturation rate	40.2(441/1098)	42.6(609/1430)	37.4(706/1887)	41.0(922/2249)	10.078	0.018
Fertilization rate	57.1(252/441)	54.5(332/609)	56.4(398/706)	59.2(546/922)	3.500	0.321
Cleavage rate	82.1(207/252)	86.1(286/332)	87.9(350/398)	85.2(465/546)	4.375	0.224
No. of transferable embryos	0.00(0.00,2.00)	1.00(0.00,3.00)	1.00(0.00,3.00)	2.00(1.00,4.75)	34.737	< 0.001
No. of high-quality embryos	0.00(0.00,1.00)	0.00(0.00,1.25)	0.50(0.00,2.00)	1.00(0.00,2.75)	30.127	< 0.001
Transferable embryo rate	38.0(114/300)	35.6(147/413)	44.1(211/479)	43.5(284/653)	9.626	0.022
High-quality embryo rate	31.9(66/207)	29.7(85/286)	38.3(134/350)	38.7(180/465)	8.626	0.035
No-available embryo cycle rate	57.6(57/99)	53.1(52/98)	46.0(46/100)	31.3(30/96)	19.813	< 0.001

### Pregnancy outcomes

Among the patients included in the study (*n* = 393, 202 (51.4%) had transferable embryos after IVM (of which 40 cycles underwent fresh embryo transfer for the first cycle, and 162 cycles underwent frozen-thawed transfer), 188 had no embryos available for transfer, and 3 patients had frozen embryos but did not proceed with transfer due to personal reasons.

Comparing pregnancy outcomes in the 202 first embryo transfer cycles, the clinical pregnancy rate in Group A was significantly higher than that in Groups B, C, and D (68.4% vs. 39.1% vs. 44.4% vs. 37.5%, *P* < 0.05). Although the live birth rate was higher than the other three groups, the difference was not statistically significant (*P* > 0.05). During the study period, there were a total of 256 transfer cycles. The cumulative clinical pregnancy rate (67.4% vs. 40.0% vs. 42.6% vs. 36.1%) and cumulative live birth rate (48.8% vs. 29.1% vs. 37.7% vs. 21.6%) in Group A were also significantly higher than those in the other three groups (*P* < 0.05) (Table 3). And the post-hoc comparisons were showed in Supplementary Table 1.

**Table 3:** Comparison of pregnancy outcomes among patients

	Group A	Group B	Group C	Group D	x^2^	*P* value
Clinical pregnancy rate following first embryo transfer	68.4(26/38)	39.1(18/46)	44.4(24/54)	37.5(24/64)	10.478	0.015
Live birth rate following first embryo transfer	47.4(18/38)	28.3(13/46)	38.9(21/54)	25.0(16/64)	6.609	0.085
Miscarriage rate following first embryo transfer	21.1(8/38)	10.9(5/46)	5.6(3/54)	12.5(8/64)	5.185	0.159
Cumulative clinical pregnancy rate	67.4(29/43)	40.0(22/55)	42.6(26/61)	36.1(35/97)	12.471	0.006
Cumulative live birth rate	48.8(21/43)	29.1(16/55)	37.7(23/61)	21.6(21/97)	11.558	0.009

Meanwhile, we also compared the pregnancy outcomes between fresh embryo transfer (*n* = 40, 19.80%) group and frozen-thawed embryo transfer (*n* = 162, 80.20%) group in the 202 first embryo transfer cycles. The results showed no statistically significant differences in clinical pregnancy rate, miscarriage rate, or live birth rate between the two groups (Supplementary Table 2).

### Multivariate logistic regression analysis of clinical pregnancy rate

Since clinical pregnancy rates following the first IVM embryo transfer cycles varied among different AMH groups, we performed multivariable logistic regression analysis to assess potential influencing factors. The results showed that compared to Group A (1 < AMH ≤ 7.77 ng/ml), Group D (AMH > 17.53 ng/ml) had a significantly lower clinical pregnancy rate (OR = 0.281, 95% CI: 0.108–0.728, *P* = 0.009). In contrast, age, basal FSH, AFC, embryo transfer type, transferred embryos and infertility causes were not significant influencing factors for the clinical pregnancy rate in the first IVM transfer cycle. (Table 4).

**Table 4:** Logistic regression analysis of clinical pregnancy rates in the first embryo transfer cycle

	B	S.E.	Wald	*P* value	OR (95% CI)
Age	-0.022	0.049	0.205	0.651	0.978(0.888–1.077)
BMI	-0.002	0.034	0.004	0.951	0.998(0.933–1.068)
Basal FSH level	0.026	0.093	0.076	0.782	1.026(0.855–1.231)
Antral follicle count	0.012	0.011	1.201	0.273	1.012(0.991–1.034)
Tubal factor	-0.059	0.404	0.022	0.883	0.942(0.427–2.081)
Ovulation dysfunction	-0.420	0.407	1.065	0.302	0.657(0.296–1.459)
AMH (A)			7.154	0.067	
AMH (B)	-0.952	0.498	3.659	0.056	0.386(0.146–1.024)
AMH (C)	-0.714	0.477	2.239	0.135	0.490(0.192–1.248)
AMH (D)	-1.270	0.486	6.829	0.009	0.281(0.108–0.728)
Embryo transfer type	-0.232	0.433	0.287	0.592	0.793(0.339–1.854)
Transferred embryos	0.209	0.189	1.222	0.269	1.232(0.851–1.784)

## Discussion

In our retrospective study, the enrolled population was divided into groups based on AMH quartiles to examine the relationship between AMH levels and IVM laboratory as well as pregnancy outcomes. We observed that Group D (AMH) > 17.53 ng/ml) demonstrated superior laboratory parameters including higher oocytes retrieved and transferable embryos compared to Group A (1 < AMH ≤ 7.77 ng/ml), while this advantage did not translate into improved clinical outcomes. Notably, Group D exhibited the lowest clinical pregnancy rates, live birth rate, cumulative clinical pregnancy rate and cumulative live birth rate among all four study groups. This indicates that within our cohort, elevated AMH and its correlated antral follicle count, while associated with increased numbers of retrieved mature oocytes, transferable embryos, and high-quality embryos, the clinical outcomes of IVM were paradoxically the poorest in this group. This suggests a discrepancy between AMH/AFC levels and IVM pregnancy outcomes.

AMH is currently one of the most reliable biomarkers for ovarian reserve and is widely used in clinical practice [24]. Higher AMH levels typically indicate better ovarian reserve, while women with polycystic ovarian morphology often exhibit elevated AMH secretion, commonly seen in PCOS [25]. As a supplement to IVF technology, IVM has now become an established ART technology, particularly for PCOS patients, as it reduces the risk of OHSS complications [26]. Nevertheless, the relationship between AMH levels and IVM pregnancy outcomes remains controversial. Our findings reveal a paradoxical association between AMH levels and treatment outcomes in IVM cycles.

Vuong’s research in Vietnam reported that live birth rates (35.5%) and cumulative ongoing pregnancy rates (44.0%) in IVM group were lower compare with IVF groups (43.5, 62.6%), but there were no statistically significant differences. Moreover, the occurrence of pregnancy complications, obstetric and perinatal complications, preterm delivery, birth weight and neonatal complications were insignificant differences in two groups [27]. However, most studies believed that IVM is associated with lower pregnancy success and reduced implantation rates compared to conventional IVF, finding that fewer than 17% of embryos derived from IVM oocytes ultimately result in clinical pregnancy [7]. IVM is not a substitute for IVF [28]. Hence, identifying optimal candidates for IVM and finding a balance between ensuring success rates and controlling OHSS risk is an ongoing clinical challenge that requires further exploration [29]. Therefore, identifying predictive markers for IVM pregnancy outcomes is crucial. One previous study suggests that the number of immature oocytes may positively correlate with IVM pregnancy outcomes [30]. Previous IVM studies in PCOS patients showed oocyte maturation rates between 40.4% and 48.0%, normal fertilization rates between 23.7% and 31.7%, and transferable embryo formation rates between 51.1% and 73.9% [31]. The number of antral follicles has been identified as an effective predictor of natural cycle oocyte retrieval numbers. Our study also found that as AMH levels increase, both AFC and the number of oocytes retrieved per cycle rise, while maturation rate, fertilization rate, and cleavage rate did not show a corresponding improvement. Moreover, the clinical pregnancy rate and live birth rate in the first IVM transfer cycle were lowest in the group with the highest AMH levels.

There is a significant correlation between AMH levels and the quantity of oocytes retrieved during IVF processes [32]. But why do the highest AMH group not achieve the best pregnancy outcomes? This may be related to the inhibitory effect of AMH on follicular growth, primarily due to its ability to reduce granulosa cell proliferation, inhibit aromatase activity, and decrease estradiol production [33–35]. It is argued that higher levels of AMH may be detrimental to ovulation outcomes as they may inhibit the recruitment of primordial follicles and reduce the ovulation rate [36]. Additionally, AMH can suppress insulin-induced follicle activation. Moreover, studies have shown that AMH inhibits FSH sensitivity and FSH receptor expression in follicles [37]. Xue Yu et al. found that AMH suppresses FSH-induced nuclear maturation of cumulus oocyte complexes (COCs), accompanied by decreased levels of MPF and *Fshr* expression, further expanding the understanding of AMH’s negative regulation of FSH-induced in vitro maturation of COCs [38]. Moreover, the vast majority of participants in our study were patients with PCOS (76.6%). Elevated AMH levels in this population may reflect more severe PCOS characteristics, such as poorer metabolic profiles and hyperandrogenism [39]. The current findings may also be related to the heterogeneity of PCOS phenotypes. In future research, we plan to further investigate differences in AMH levels and pregnancy outcomes among patients with distinct PCOS phenotypes.

The study by Hyun Ha et al. [40] indicated that serum AMH levels were an effective predictor of pregnancy outcomes for PCOS patients prior to initiating IVM cycles. With AMH levels at or above 8.5 ng/ml, the clinical pregnancy rates, ongoing pregnancy rates, miscarriage rates, and live birth rates during IVM, were comparable to those observed with traditional IVF treatments, with no instances of ovarian hyperstimulation syndrome reported during the IVM cycles. Research by Mostinckx L et al. has also revealed a positive correlation between serum AMH levels and cumulative ongoing pregnancy rates following IVM treatment [41]. In infertile women undergoing ART, IVM and ovarian stimulation demonstrated similar reproductive success rates among patients with AMH levels ≥10 ng/ml. However, due to inherent selection bias, the reliability of these findings diminishes at the higher end of the AMH spectrum. In our study, we found that Group A (1 < AMH ≤ 7.77 ng/mL) exhibited the most favorable pregnancy outcomes, suggesting that not all patients with PCOS or high responders may be suitable candidates for IVM. IVM may offer greater advantages for individuals with relatively normal ovarian responses. In the future, comparing pregnancy outcomes between IVM and IVF in patients with similar AMH levels during the same period could provide more helpful and clinically relevant insights.

IVM, possibly due to the absence of the hCG trigger, leads to poor endometrial receptivity, resulting in lower pregnancy success rates with fresh embryo transfers. Previous studies indicate that freezing all embryos can improve pregnancy outcomes in IVM, with clinical pregnancy rates around 24% per IVM cycle [42]. A randomized controlled study among infertile patients aged 18–37 with more than 24 antral follicles (*n* = 40) showed that the ongoing pregnancy rates (65% vs. 25%, *P* = 0.03) and live birth rates (60% vs. 20%, *P* = 0.02) were significantly higher in the frozen-thawed embryo transfer group post-IVM compared to the fresh embryo transfer group. Clinical pregnancy rates were higher in the frozen-thawed group compared to the fresh group, this increase did not reach statistical significance (70% vs. 35%, *P* = 0.06) [43]. Despite studies indicating lower pregnancy success rates for fresh embryo transfers post-IVM [29], there has been an increase in patients choosing fresh embryo transfers post-IVM recently. Our analysis indicated no marked differences between fresh post-IVM embryo transfers and first-time thawed embryo transfers concerning clinical pregnancy rates (45.5% vs. 50.9%), miscarriage rates (12.1% vs. 12.6%), and live birth rates (33.3% vs. 36.6%) (*P* > 0.05). This discrepancy may be attributed to the fact that only a small proportion of IVM cycles permitted fresh embryo transfer, combined with variations in IVM and endometrial preparation protocols across centers.

IVM has shown significant potential for application in selected patients or scenarios. However, due to the limited understanding of the molecular and metabolic mechanisms underlying oocyte maturation, as well as the substantial quality gap between in vitro-matured and in vivo-matured oocytes, the clinical pregnancy rate of IVM remains considerably lower than that of conventional IVF. The primary obstacle to the widespread adoption of IVM lies in the suboptimal culture conditions for human oocyte maturation in vitro, which encompasses both the refinement of culture medium formulations and the improvement of oocyte manipulation and culture techniques. Recent advances in basic research, bioengineering, and materials science—particularly the development of strategies such as stimulated physiological oocyte maturation (SPOM) or biphasic IVM, along with the introduction of dynamic culture systems and biomaterial technologies - have enabled better simulation of the physiological follicular microenvironment [44]. These innovations hold promise for enhancing the developmental competence of oocytes, improving clinical outcomes, and thereby expanding the prospects for IVM.

The current study has several limitations. First, as a retrospective study, our research is subject to the influence of confounding factors, such as varying causes of infertility. Notably, our cohort was not exclusively composed of PCOS patients, with PCOS individuals accounting for 76.6%, and the potential impact of PCOS phenotypes was not considered. Future analyses should be conducted in homogeneous PCOS populations after adjusting for relevant confounding factors. Secondly, while previous studies have demonstrated an increased risk of preterm birth following embryo transfer in PCOS patients with elevated AMH levels [45], our study did not assess other important pregnancy complications including preterm birth, gestational diabetes mellitus, or pregnancy-induced hypertension. Thirdly, the relatively small sample size may limit the statistical power of our analyses, highlighting the need for larger-scale, prospective clinical studies to validate these findings.

In summary, our findings demonstrate that while IVM yields sufficient oocyte and embryo numbers in patients with high AMH (AMH > 17.53 ng/mL), the optimal clinical outcomes were achieved within an intermediate AMH range (1-7.77 ng/mL) despite suboptimal laboratory parameters. These results underscore the clinical value of AMH-stratified IVM protocols. Future multicenter studies should investigate the molecular mechanisms underlying AMH-associated oocyte dysfunction and establish evidence-based AMH cut-off values for personalized IVM treatment strategies. The future development of IVM will rely on the deep integration of molecular biology, engineering technology, and clinical research. It is believed that with the advancement of scientific and technological capabilities across various fields, IVM has the potential to become a safe, effective, and routinely applicable assisted reproductive technology.

## Supplementary Information

Supplementary Material 1.

## Data Availability

The data that support the findings of this study are available on request from the corresponding author. The data are not publicly available due to privacy or ethical restrictions.

## References

[1] Sermondade N, Grynberg M, Comtet M, et al. Double-in vitro maturation increases the number of vitrified oocytes available for fertility preservation when ovarian stimulation is unfeasible [J]. Sci Rep. 2020;10(1):18555. 33122722 10.1038/s41598-020-75699-x PMC7596087

[2] Cha KY, Ko JJ, Choi DH, et al. Pregnancy after in vitro fertilization of human follicular oocytes collected from nonstimulated cycles, their culture in vitro and their transfer in a donor oocyte program [J]. Fertil Steril. 1991;55(1):109–13. 1986950 10.1016/s0015-0282(16)54068-0

[3] Sauerbrun-Cutler MT, Vega M, Keltz M, et al. In vitro maturation and its role in clinical assisted reproductive technology [J]. Obstet Gynecol Surv. 2015;70(1):45–57. 10.1097/OGX.0000000000000150. 25616347 10.1097/OGX.0000000000000150

[4] [4] Braam SC, Ho VNA, Pham TD, et al. In-vitro maturation versus IVF: a cost-effectiveness analysis [J]. Reprod Biomed Online. 2021;42(1):143–9. 10.1016/j.rbmo.2020.09.022. 33132059 10.1016/j.rbmo.2020.09.022

[5] Walls ML, Hart RJ. In vitro maturation [J]. Best Pract Res Clin Obstet Gynaecol. 2018;53:60–72. 10.1016/j.bpobgyn.2018.06.004. 30056110 10.1016/j.bpobgyn.2018.06.004

[6] Practice Committees of the American Society for Reproductive Medicine. The society of reproductive biologists and Technologists, and the society for assisted reproductive Technology. In vitro maturation: a committee opinion [J]. Fertil Steril. 2021;115(2):298–304. 10.1016/j.fertnstert.2020.11.018. 33358333 10.1016/j.fertnstert.2020.11.018

[7] Galvão A, Segers I, Smitz J, et al. In vitro maturation (IVM) of oocytes in patients with resistant ovary syndrome and in patients with repeated deficient oocyte maturation [J]. J Assist Reprod Genet. 2018;35(12):2161–71. 10.1007/s10815-018-1317-z. 30238176 10.1007/s10815-018-1317-z PMC6289928

[8] Lv Y, Wu S, Miao M, et al. Success of oocyte retrieval in modified natural cycle assisted reproductive techniques: a retrospective cohort study [J]. J Obstet Gynaecol. 2026;46(1):2579425. 10.1080/01443615.2025.2579425. 41518116 10.1080/01443615.2025.2579425

[9] Huang X, Chen J, Yu Y, et al. The impact of polycyclic aromatic hydrocarbon metabolites on reproductive hormone levels and follicle count in patients with polycystic ovary syndrome: a case-control study [J]. Int J Environ Health Res. 2025;35(12):3977–88. 10.1080/09603123.2025.2506136. 40376704 10.1080/09603123.2025.2506136

[10] Jung SA-O, Allen N, Arslan AA, et al. Anti-Müllerian hormone and risk of ovarian cancer in nine cohorts [J]. Int J Cancer. 2018;142(2):262–70. 10.1002/ijc.31058. 28921520 10.1002/ijc.31058 PMC5749630

[11] Zhang MA-O, Feng J, Ma W, et al. Current status of fertility preservation procedures in gynecologic oncology: from a Chinese perspective [J]. J Assist Reprod Genet. 2025;42(2):635–45. 10.1007/s10815-024-03341-0. 39812764 10.1007/s10815-024-03341-0 PMC11871211

[12] Bunyaeva E, Kirillova A, Khabas G, et al. Feasibility of in vitro maturation of oocytes collected from patients with malignant ovarian tumors undergoing fertility preservation [J]. Int J Gynecol Cancer. 2021;31(3):475–9. 10.1136/ijgc-2020-001754. 33649016 10.1136/ijgc-2020-001754

[13] Buratini JA-O, Dellaqua TT, Dal Canto M, et al. The putative roles of FSH and AMH in the regulation of oocyte developmental competence: from fertility prognosis to mechanisms underlying age-related subfertility [J]. Hum Reprod Update. 2022;28(2):232–54. 10.1093/humupd/dmab044. 34969065 10.1093/humupd/dmab044

[14] Nelson SA-O, Davis S, A-O, Kalantaridou S, A-O, et al. Anti-Müllerian hormone for the diagnosis and prediction of menopause: a systematic review [J]. Hum Reprod Update. 2023;29(3):327–46. 10.1093/humupd/dmac045. 36651193 10.1093/humupd/dmac045 PMC10152172

[15] Xu H, Zhang M, Zhang H, et al. Clinical applications of serum anti-Müllerian hormone measurements in both males and females: an update [J]. Innov (Camb). 2021;2(1):100091. 10.1016/j.xinn.2021.100091. 10.1016/j.xinn.2021.100091 PMC8454570 34557745

[16] Ma C, Xu H, Wang H, et al. An online tool for predicting ovarian responses in unselected patients using dynamic inhibin B and basal antimüllerian hormone levels [J]. Front Endocrinol (Lausanne). 2023;20(14):1074347. 10.3389/fendo.2023.1074347. 10.3389/fendo.2023.1074347 PMC9895413 36742391

[17] Xu H, Feng G, Alpadi K, et al. A model for predicting polycystic ovary syndrome using serum AMH, menstrual cycle Length, body mass index and serum Androstenedione in Chinese reproductive aged population: A retrospective cohort study [J]. Front Endocrinol (Lausanne). 2022;17(13):821368. 10.3389/fendo.2022.821368. 10.3389/fendo.2022.821368 PMC8970043 35370993

[18] Yuwen T, Yang Z, Cai G, et al. Association between serum AMH levels and IVF/ICSI outcomes in patients with polycystic ovary syndrome: a systematic review and meta-analysis [J]. Reprod Biol Endocrinol. 2023;21(1):95. 10.1186/s12958-023-01153-y. 37872575 10.1186/s12958-023-01153-y PMC10591359

[19] Seok HH, Song H, Lyu SW, et al. Application of serum anti-Müllerian hormone levels in selecting patients with polycystic ovary syndrome for in vitro maturation treatment [J]. Clin Exp Reprod Med. 2016;43(2):126–32. 10.5653/cerm.2016.43.2.126. 27358832 10.5653/cerm.2016.43.2.126 PMC4925868

[20] Ito K, Takae S, Nakamura K, et al. The study of the efficiency of in vitro maturation of ovarian tissue oocytes in pediatric patients [J]. J Assist Reprod Genet. 2023;40(12):2787–97. 10.1007/s10815-023-02958-x. 37779181 10.1007/s10815-023-02958-x PMC10656375

[21] Zheng X, Guo W, Zeng L, et al. In vitro maturation without gonadotropins versus in vitro fertilization with hyperstimulation in women with polycystic ovary syndrome: a non-inferiority randomized controlled trial [J]. Hum Reprod. 2022;37(2):242–53. 10.1093/humrep/deab243. 34849920 10.1093/humrep/deab243 PMC9115328

[22] Alpha Scientists in Reproductive Medicine and ESHRE Special Interest Group of Embryology. The Istanbul consensus workshop on embryo assessment: proceedings of an expert meeting [J]. Hum Reprod Open. 2011; 26(6): 1270–1283. 10.1093/humrep/der037 21502182

[23] Gardner DK, L M. Blastocyst score affects implantation and pregnancy outcome: towards a single blastocyst transfer [J]. Fertil Steril. 2000;73(6):1155–8. 10856474 10.1016/s0015-0282(00)00518-5

[24] Colaco S, A-O, Achrekar SA-O, Patil A, et al. Association of AMH and AMHR2 gene polymorphisms with ovarian response and pregnancy outcomes in Indian women [J]. J Assist Reprod Genet. 2022;39(7):1633–42. 10.1007/s10815-022-02541-w. 35713750 10.1007/s10815-022-02541-w PMC9365902

[25] Moolhuijsen L, Louwers Y, Laven J, et al. Comparison of 3 different AMH assays with AMH levels and follicle count in women with polycystic ovary syndrome [J]. J Clin Endocrinol Metab. 2022;107(9):e3714–22. 10.1210/clinem/dgac370. 35737957 10.1210/clinem/dgac370 PMC9387710

[26] Vuong LN, Ho VNA, Le AH, et al. Hormone-free vs. follicle-stimulating hormone-primed infertility treatment of women with polycystic ovary syndrome using biphasic in vitro maturation: a randomized controlled trial [J]. Fertil Steril. 2025;123(2):253–61. 10.1016/j.fertnstert.2024.09.010. 39260537 10.1016/j.fertnstert.2024.09.010

[27] Vuong LN, Ho VNA, Ho TM, et al. In-vitro maturation of oocytes versus conventional IVF in women with infertility and a high antral follicle count: a randomized non-inferiority controlled trial [J]. Hum Reprod. 2020;35(11):2537–47. 10.1093/humrep/deaa240. 32974672 10.1093/humrep/deaa240

[28] Mostinckx L, Segers I, Belva F, et al. Obstetric and neonatal outcome of ART in patients with polycystic ovary syndrome: IVM of oocytes versus controlled ovarian stimulation [J]. Hum Reprod. 2019;34(8):1595–607. 10.1093/humrep/dez086. 31347678 10.1093/humrep/dez086

[29] Walls ML, Hunter T, Ryan JP, et al. In vitro maturation as an alternative to standard in vitro fertilization for patients diagnosed with polycystic ovaries: a comparative analysis of fresh, frozen and cumulative cycle outcomes [J]. Hum Reprod. 2015;30(1):88–96. 10.1093/humrep/deu248. 25355587 10.1093/humrep/deu248

[30] Guzman L, O-H C. P. P N, A prediction model to select PCOS patients suitable for IVM treatment based on anti-Mullerian hormone and antral follicle count [J]. Human reproduction (Oxford, England), 2013; 28(5): 1261–6. 10.1093/humrep/det034 23427238

[31] Liu T, Liu D, Song X, et al. Relationship between the number of oocytes retrieved and in vitro maturation outcomes in patients with polycystic ovary syndrome undergoing natural cycles [J]. Chin J Reprod Contracept. 2022;42(8):782–90. 10.3760/cma.j.cn101441-20210306-00102.

[32] Sun XY, Lan YZ, Liu S et al. Relationship Between Anti-Müllerian Hormone and In Vitro Fertilization-Embryo Transfer in Clinical Pregnancy [J]. Front Endocrinol (Lausanne), 2020;11(595448). 10.3389/fendo.2020.595448 PMC7746804 33343511

[33] Grossman MP, Nakajima St Fau - Fallat ME, Fallat Me Fau - Siow Y, et al. Müllerian-inhibiting substance inhibits cytochrome P450 aromatase activity in human granulosa lutein cell culture [J]. Fertil Steril. 2008;89(5 Suppl):1364–70. 10.1016/j.fertnstert.2007.03.066. 17517397 10.1016/j.fertnstert.2007.03.066

[34] Chang HM, Klausen C, Fau - Leung PCK, Leung PC. Antimüllerian hormone inhibits follicle-stimulating hormone-induced adenylyl cyclase activation, aromatase expression, and estradiol production in human granulosa-lutein cells [J]. Fertil Steril. 2013;100(2):585–92. 10.1016/j.fertnstert.2013.04.019. 23663993 10.1016/j.fertnstert.2013.04.019

[35] Tsai HW, Liao PF, Li CJ, et al. High serum anti-Müllerian hormone concentrations have a negative impact on fertilization and embryo development rates [J]. Reprod Biomed Online. 2022;44(1):171–6. 10.1016/j.rbmo.2021.08.016. 34801403 10.1016/j.rbmo.2021.08.016

[36] Vagios S, Sacha CR, Hammer KC, et al. Response to ovulation induction treatments in women with polycystic ovary syndrome as a function of serum anti-Müllerian hormone levels [J]. J Assist Reprod Genet. 2021;38(7):1827–33. 33934267 10.1007/s10815-021-02217-x PMC8324640

[37] Pellatt L, Rice S, Fau - Dilaver N, Dilaver N, Fau - Heshri A, et al. Anti-Müllerian hormone reduces follicle sensitivity to follicle-stimulating hormone in human granulosa cells [J]. Fertil Steril. 2011;96(5):1246–51. 10.1016/j.fertnstert.2011.08.015. 21917251 10.1016/j.fertnstert.2011.08.015

[38] Yu X, Li Z, Zhao X et al. Anti-Müllerian Hormone Inhibits FSH-Induced Cumulus Oocyte Complex In Vitro Maturation and Cumulus Expansion in Mice. LID – 10.3390/ani12091209 [doi] LID – 1209 [J]. Animals (Basel), 2022; 12(9): 1209. 10.3390/ani12091209. 10.3390/ani12091209 PMC9103408 35565634

[39] Delamuta LC, Fassolas G, Dias Júnior JA, et al. Antimüllerian hormone levels and IVF outcomes in polycystic ovary syndrome women: a scoping review [J]. JBRA Assist Reprod. 2024;28(2):299–305. 10.5935/1518-0557.20230059. 38446747 10.5935/1518-0557.20230059 PMC11152416

[40] Seok HH, Song H, Lyu SW, et al. Application of serum anti-Müllerian hormone levels in selecting patients with polycystic ovary syndrome for in vitro maturation treatment [J]. Clin Exp Reprod Med. 2016;43(2):126–32. 27358832 10.5653/cerm.2016.43.2.126 PMC4925868

[41] Mostinckx L, Goyens E, Mackens S, et al. Clinical outcomes from ART in predicted hyperresponders: in vitro maturation of oocytes versus conventional ovarian stimulation for IVF/ICSI [J]. Hum Reprod. 2024;39(3):586–94. 10.1093/humrep/dead273. 38177084 10.1093/humrep/dead273

[42] Ortega-Hrepich C. A freeze-all embryo strategy after in vitro maturation: a novel approach in women with polycystic ovary syndrome? [J]. Fertil Steril. 2013;100(4):1002–7. 23850301 10.1016/j.fertnstert.2013.06.018

[43] Vuong LA, Nguyen LK, Le AH, et al. Fresh embryo transfer versus freeze-only after in vitro maturation with a pre-maturation step in women with high antral follicle count: a randomized controlled pilot study [J]. J Assist Reprod Genet. 2021;38(6):1293–302. 33825118 10.1007/s10815-021-02180-7 PMC8266980

[44] Gargallo-Alonso M, Picton HM, Malo C, Advances. Mechanisms, and clinical perspectives for the in vitro maturation of human Oocytes. LID – 10.3390/ijms27010005 [doi] LID – 5 [J]. Int J Mol Sci. 2025;27(1):5. 10.3390/ijms27010005. 41515886 10.3390/ijms27010005 PMC12785462

[45] Hu KL, Liu FT, Xu H, et al. High antimüllerian hormone levels are associated with preterm delivery in patients with polycystic ovary syndrome [J]. Fertil Steril. 2020;113(2):444–52. 31973904 10.1016/j.fertnstert.2019.09.039

