# Supplementary material for: Impact of anti-Müllerian hormone on pregnancy outcomes in in vitro maturation: a retrospective cohort study
Source: J Ovarian Res. 2026 Feb 25;19:126. doi: 10.1186/s13048-026-02033-w (PMC13041493; doi:10.1186/s13048-026-02033-w)
Supplement: Supplementary file 1 — Supplementary Material 1. [file 13048_2026_2033_MOESM1_ESM.docx]

**Supplemental Table1 *P*-values for pairwise comparisons of significantly different variables**

| Variables | Group A vs. B | Group A vs. C | Group A vs. D | Group B vs C | Group B vs. D | Group C vs. D |
| --- | --- | --- | --- | --- | --- | --- |
| Basal FSH levels | 0.950 | 0.638 | 0.011 | 0.586 | 0.008 | 0.033 |
| Antral follicle count | 0.027 | 0.000 | 0.000 | 0.017 | 0.000 | 0.053 |
| Ovulatory dysfunction | 0.076 | 0.000 | 0.000 | 0.070 | 0.014 | 0.484 |
| Tubal factor | 0.962 | 0.094 | 0.025 | 0.086 | 0.022 | 0.547 |
| No. of oocytes retrieved | 0.001 | 0.000 | 0.000 | 0.018 | 0.000 | 0.029 |
| Maturation rate | 0.220 | 0.136 | 0.646 | 0.003 | 0.340 | 0.019 |
| No. of transferable embryos | 0.067 | 0.002 | 0.000 | 0.186 | 0.000 | 0.010 |
| No. of high-quality embryos | 0.119 | 0.001 | 0.000 | 0.102 | 0.000 | 0.039 |
| No available embryo cycle rate | 0.524 | 0.102 | 0.000 | 0.320 | 0.002 | 0.034 |
| Embryo development rate | 0.510 | 0.096 | 0.110 | 0.010 | 0.010 | 0.852 |
| High-quality embryo rate | 0.607 | 0.128 | 0.090 | 0.024 | 0.012 | 0.902 |
| Clinical pregnancy rate following first embryo transfer | 0.007 | 0.023 | 0.003 | 0.592 | 0.862 | 0.444 |
| Cumulative clinical pregnancy rate | 0.007 | 0.013 | 0.001 | 0.775 | 0.632 | 0.411 |
| Cumulative live birth rate | 0.045 | 0.258 | 0.001 | 0.327 | 0.304 | 0.028 |

Statistical significance: *P* < 0.008.

**Supplemental Table2 Comparison of pregnancy outcomes in patients during first fresh versus frozen-thawed embryo transfer cycles**

|  | Fresh cycles（n=40） | frozen-thawed cycles（n=162） | *x*^2^ | *P value* |
| --- | --- | --- | --- | --- |
| Clinical pregnancy rate | 42.5(17/40) | 46.3(75/162) | 0.186 | 0.666 |
| Miscarriage rate | 12.5(5/40) | 11.7(19/162) | 0.018 | 0.893 |
| Live birth rate | 30.0(12/40) | 34.6(56/162) | 0.300 | 0.584 |
